# Crystal structure of dihydrodipicolinate reductase (*Pa*DHDPR) from *Paenisporosarcina* sp. TG-14: structural basis for NADPH preference as a cofactor

**DOI:** 10.1038/s41598-018-26291-x

**Published:** 2018-05-21

**Authors:** Chang Woo Lee, Sun-Ha Park, Sung Gu Lee, Hyun Ho Park, Hak Jun Kim, HaJeung Park, Hyun Park, Jun Hyuck Lee

**Affiliations:** 1 Unit of Polar Genomics, Korea Polar Research Institute, Incheon, 21990 Republic of Korea; 2 Department of Polar Sciences, University of Science and Technology, Incheon, 21990 Republic of Korea; 3 College of Pharmacy, Chung-Ang University, 84 Heukseok-ro, Dongjak, Seoul, 06974 Republic of Korea; 4 Department of Chemistry, Pukyong National University, 45 Yongso-ro, Busan, 48513 Republic of Korea; 5 X-Ray Core, TRI, The Scripps Research Institute, Jupiter, FL 33458 USA

## Abstract

Dihydrodipicolinate reductase (DHDPR) is a key enzyme in the diaminopimelate- and lysine-synthesis pathways that reduces DHDP to tetrahydrodipicolinate. Although DHDPR uses both NADPH and NADH as a cofactor, the structural basis for cofactor specificity and preference remains unclear. Here, we report that *Paenisporosarcina* sp. TG-14 *Pa*DHDPR has a strong preference for NADPH over NADH, as determined by isothermal titration calorimetry and enzymatic activity assays. We determined the crystal structures of *Pa*DHDPR alone, with its competitive inhibitor (dipicolinate), and the ternary complex of the enzyme with dipicolinate and NADPH, with results showing that only the ternary complex had a fully closed conformation and suggesting that binding of both substrate and nucleotide cofactor is required for enzymatic activity. Moreover, NADPH binding induced local conformational changes in the N-terminal long loop (residues 34–59) of *Pa*DHDPR, as the His35 and Lys36 residues in this loop interacted with the 2′-phosphate group of NADPH, possibly accounting for the strong preference of *Pa*DHDPR for NADPH. Mutation of these residues revealed reduced NADPH binding and enzymatic activity, confirming their importance in NADPH binding. These findings provide insight into the mechanism of action and cofactor selectivity of this important bacterial enzyme.

## Introduction

Sporulation is a major survival strategy for certain Gram-positive bacteria, including *Bacillus* and *Clostridium* species^1–3^. Endospore formation begins with asymmetrical cell division, followed by the formation of an outer cell wall comprising various coat proteins that enable bacteria to resist harsh environmental stress. Over 500 genes are involved in sporulation^4–6^, and dihydrodipicolinate (DHDP) is a key compound in bacterial sporulation that acts as a precursor for two important pathways: the dipicolinate (DPA)-synthesis pathway and the diaminopimelate (DAP)- and lysine-synthesis pathway^7–10^. DPA is a major component of bacterial endospores (5–15% of the total mass) that forms a complex with calcium ions and causes spore dehydration by binding free water molecules^11^. The calcium-DPA complex also protects bacterial DNA against environmental stresses by inserting itself between nucleotides^12,13^. On the other hand, DAP is a component of peptidoglycan in the cell wall of many bacteria that forms cross-links with and thereby confers rigidity to the bacterial cell wall^14–16^. These two pathways mutually regulate cellular activity through a feedback mechanism, with DPA inhibiting lysine synthesis^17,18^. Moreover, enzymes in these two pathways are potential targets for the design of antibiotics against spore-forming pathogens.

DHDP reductase (DHDPR; *dapB*) is a branching enzyme associated with two described pathways and that reduces DHDP to tetrahydroDPA using NAD(P)H as a cofactor^19^. To date, crystal structures of DHDPR from *Escherichia coli*, *Mycobacterium tuberculosis*, *Thermotoga maritima*, *Staphylococcus aureus*, *Bartonella henselae*, and *Corynebacterium glutamicum* have been resolved^20–25^. DHDPR has two domains, including an N-terminal nucleotide-binding domain and a C-terminal domain for substrate binding and tetramerization^20^. Cofactor and substrate binding induce the switch between the open and closed conformations of these domains. The preference of DHDPR for NADH or NADPH varies across species, with orthologues in *E. coli* (*Ec*DHDPR) and *M. tuberculosis* (*Mt*DHDPR) preferring NADH over NADPH, whereas the opposite is true for the *T. maritima* and *S. aureus* orthologues (*Tm*DHDPR and *Sa*DHDPR, respectively)^21,23,26–28^. However, the mechanisms associated with DHDPR discrimination between NADH and NADPH and the region and residues responsible for cofactor selectivity remain unknown.

To address these issues, we determined the structure and characterized the biochemical properties of DHDPR from the psychrophilic bacterium *Paenisporosarcina* sp. TG-14 (*Pa*DHDPR) isolated from sediment-laden, stratified basal ice from Taylor glacier, McMurdo dry valley, Antarctica^29^. This Gram-positive species is reportedly capable of surviving millions of years in the spore state^30–32^. The results of isothermal titration calorimetry (ITC) and enzyme activity assays demonstrated that *Pa*DHDPR has a strong preference for NADPH as a cofactor. Additionally, we determined the crystal structures of unliganded *Pa*DHDPR and the enzyme in complex with DPA alone or with NADPH and performed mutagenesis studies in order to gain insight into the structural basis for this cofactor preference.

## Results and Discussion

### Biochemical characterization of PaDHDPR

**Figure 1:**
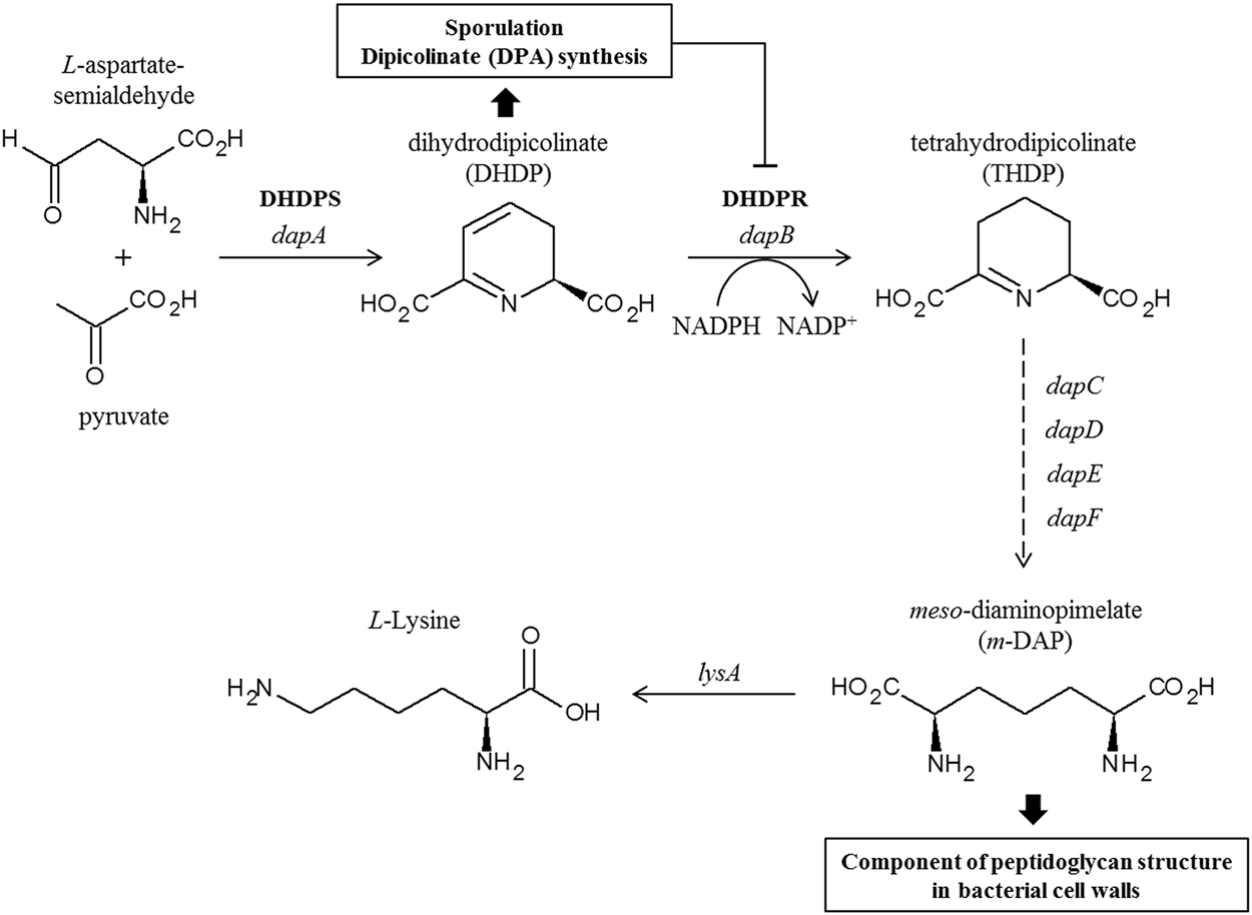
Schematic representation of reactions catalysed by DHDPS and DHDPR in the lysine-biosynthesis pathway. DHDP is a precursor for two separate pathways: the DPA (a major component of bacterial spores)-synthesis pathway and lysine-synthesis pathway. DHDPR catalyses the reduction of DHDP into tetrahydrodipicolinate (THDP) using NAD(P)H as a cofactor.

**Figure 2:**
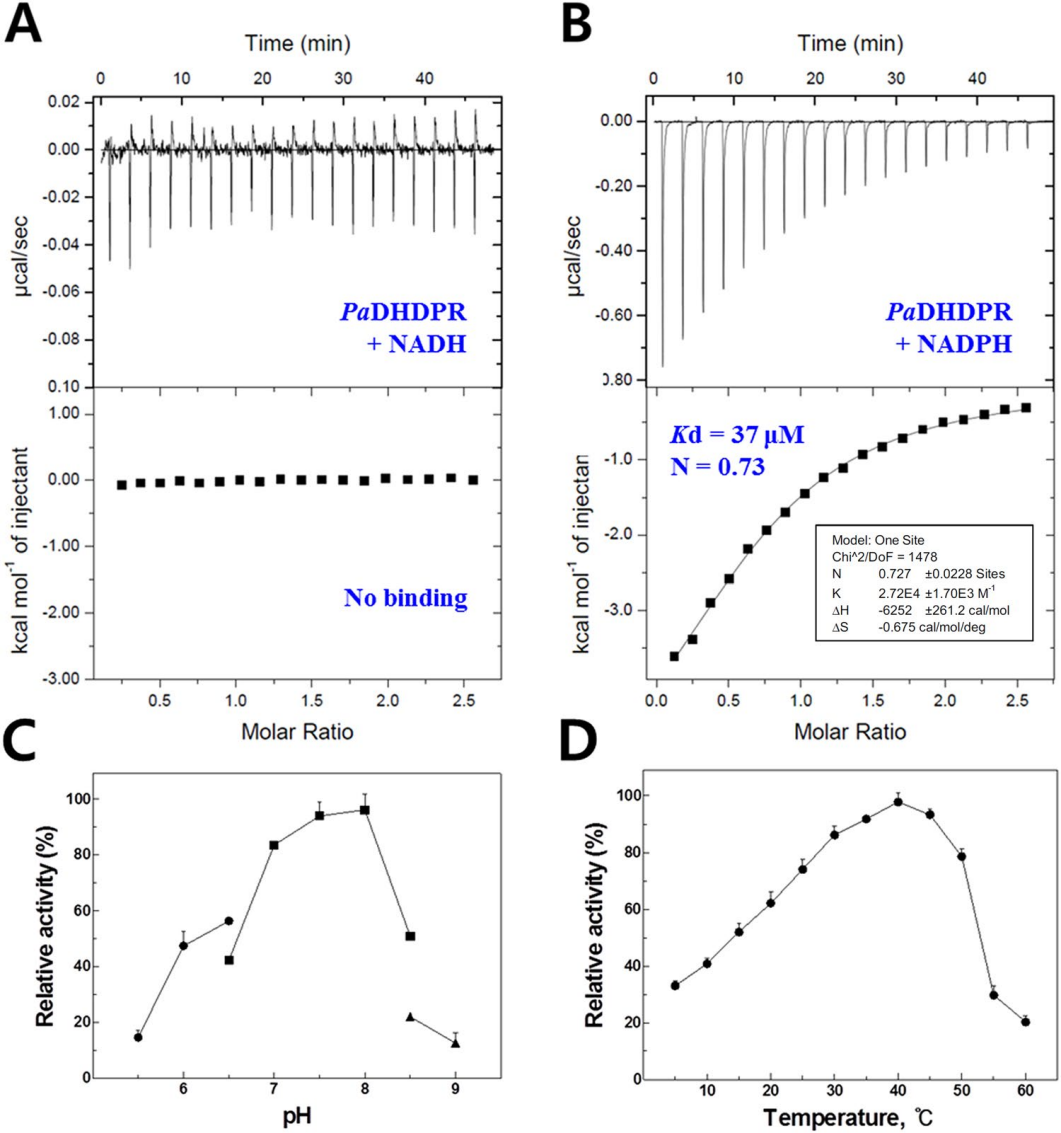
Biochemical properties of *Pa*DHDPR. ITC measurements of *Pa*DHDPR with (**A**) NADH and (**B**) NADPH. Original raw data (top panel) and the fit after integration (bottom panel) are shown. Data were fitted using a one-site binding model. Parameters obtained from curve-fitting for NADPH are as follows: ΔS = −0.675 cal mol^−1^deg^−1^, ΔH = −6.25 kcal mol^−1^, *K*_d_ = 0.07 μM, and n = 0.73. (**C**) Effect of pH on enzyme activity. Relative activity was determined in 100 mM sodium acetate (pH 5.0–6.5) (●), 100 mM HEPES (pH 6.5–8.0) (■), and 100 mM Tris-HCl (pH 8.0–9.0) (▲) at 25 °C. Activity at the optimal pH was set as 100%. (**D**) Effect of temperature on enzyme activity. Relative activity at different temperatures (5–60 °C) was determined in 100 mM HEPES buffer (pH 7.5). Activity at the optimal temperature was set as 100%. All measurements were performed in triplicate. The error bar indicates the standard deviation.

DHDPR uses nucleotides as an electron source (Fig. 1), with nucleotides bound to the positively charged nucleotide-binding site at the N-terminus serving as a cofactor. The *k*_cat_ value for *Pa*DHDPR was 7.7-fold higher in the presence of NADPH, whereas the *K*_m_ was 2-fold lower for NADPH than that for NADH, demonstrating that the enzyme strongly prefers the phosphorylated form of the molecule (NADPH). To confirm this result, we evaluated the binding affinities of NADH and NADPH by ITC (Fig. 2A,B). Titration experiments were performed with 80 μM *Pa*DHDPR in the sample cell and 1 mM NADH or NADPH. NADH did not bind to *Pa*DHDPR; however, *Pa*DHDPR had strong affinity for NADPH, with a *K*_d_ value of 37 μM (Fig. 2A), which was slightly lower than those reported for *Ec*DHDPR (2.1 μM) and *Sa*DHDPR (0.9 μM)^23,26,28^.

*Tm*DHDPR and *Sa*DHDPR are inhibited by high substrate concentrations^27,28^. When l-aspartate-semialdehyde (ASA) concentrations were varied using NADPH as a cofactor, the *k*_cat_ and *K*_m_ of *Pa*DHDPR were 52 ± 1.1 s^−1^ and 12 ± 1.6 μM, respectively (Supplementary Table S1). Notably, *Pa*DHDPR showed no inhibition at concentrations up to 0.2 mM ASA, which was consistent with results for *Ec*DHDPR and *Mt*DHDPR^23,26^. This suggests that the lysine-synthesis pathway is differentially regulated depending on the species. We then investigated the melting temperature, pH dependence, and optimal temperature of *Pa*DHDPR using NADPH as a cofactor (Fig. 2A,D). The melting temperature of *Pa*DHDPR was 52.7 °C (Supplementary Fig. S1), and maximal activity was observed at a pH range of 7.5 to 8.0, which is similar to the optimal pH of the *Bacillus* homolog, and 40 °C^33^. Although the recombinant *Pa*DHDPR enzyme did not exhibit a low optimal temperature expected for enzymes isolated from psychrophilic bacteria, >60% of the maximal activity was retained at 20 °C. Some multi-subunit enzymes show characteristics of thermal stability similar to mesophilic homologues, despite their having been isolated from psychrophilic organisms^34,35^. A previous study showed that maize DHDPR is highly sensitive to heat, and that its activity is sharply reduced under such conditions (a 69% loss of activity at 45 °C)^36^. Recent studies also suggested that plant DHDPRs might have different quaternary structure as a dimer according to investigation of *Arabidopsis thaliana* DHDPR^37^. These findings suggest that bacterial DHDPRs are more heat stable than plant DHDPRs due to their forming a tetramer in solution (Supplementary Fig. S1).

### Crystal structure of PaDHDPR

**Figure 3:**
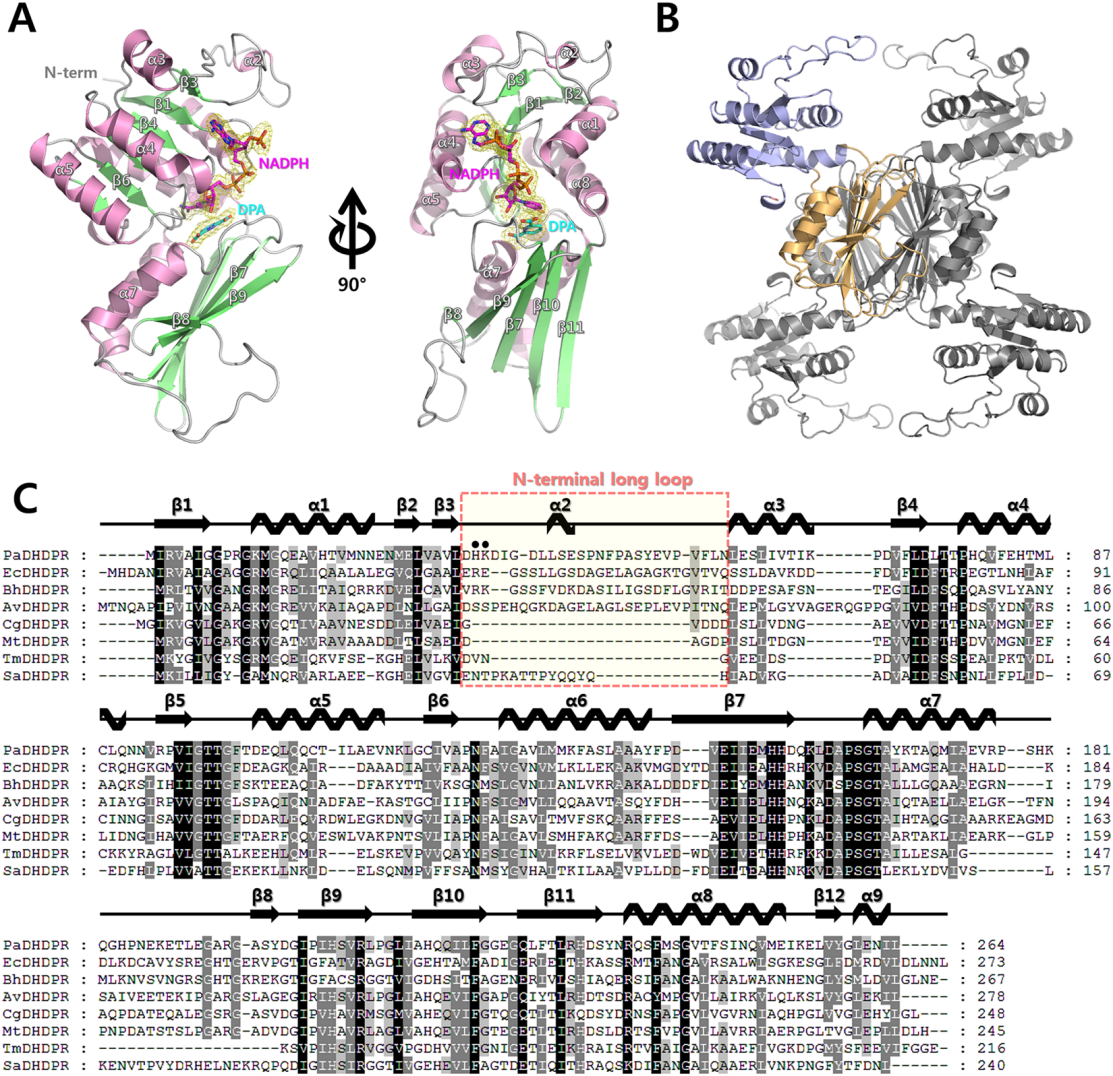
Crystal structure of *Pa*DHDPR and multiple sequence alignment with other DHDPRs. (**A**) Overall fold of *Pa*DHDPR, showing α-helices in pink and β-strands in lime. Bound NADPH (magenta) and DPA (cyan) are shown as stick models. The 2Fo-Fc electron density map (contoured at 1.0-δ level; yellow) around NADPH and DPA are shown as a mesh. (**B**) *Pa*DHDPR forms a tetramer through its C-terminal domain (light orange). The *Pa*DHDPR monomer includes N-terminal nucleotide-binding (light blue) and C-terminal substrate-binding (light orange) domains. (**C**) Multiple sequence alignment of representative DHDPRs, including sequences of homologs from *E. coli* (*Ec*DHDPR; UniProtKB: P04036; PDB: 1DRU), *B. henselae* (*Bh*DHDPR; UniProtKB: Q6G2G3; PDB: 3IJP), *A. variabilis* (*Av*DHDPR; UniProtKB: Q3MFY8; PDB: 5KT0), *T. maritima* (*Tm*DHDPR; UniProtKB: Q9X1X6; PDB: 1H2H), *S. aureus* (*Sa*DHDPR; UniProtKB: Q5HG24; PDB: 3QY9), *C. glutamicum* (*Cg*DHDPR; UniProtKB: P40110; PDB: 5EER), and *M. tuberculosis* (*Mt*DHDPR; UniProtKB: P9WP23; PDB: 1YL5. NLL regions (residues 34–59) are boxed in salmon and shaded in yellow. The His35 and Lys36 residues (filled circles) form strong electrostatic interactions with the 2′-phosphate group of NADPH. Corresponding secondary structures are shown above the sequence.

The crystal structures of apo *Pa*DHDPR (without the cofactor) and the DPA- and NADPH/DPA-bound forms of the enzyme were determined at 1.8 Å, 1.8 Å, and 2.1 Å resolutions, respectively (Fig. 3). Despite *Pa*DHDPR sharing 42% sequence identity with DHDPR from *Anabaena variabilis* [*Av*DHDPR; Protein Data Bank (PDB): 5KT0, unpublished], molecular replacement did not yield a solution. Therefore, an initial partial structure of *Pa*DHDPR was determined at 2.0 Å by single-wavelength anomalous dispersion (SAD) based on the anomalous scattering of Se atoms^38^. The final 1.8 Å high-resolution native model of *Pa*DHDPR was refined and obtained by molecular replacement using the initial SAD-phasing model. Both were crystallized in the hexagonal space group *P*6_4_22, with one molecule in the asymmetric unit. *Pa*DHDPR tetramerization was observed by analytical centrifugation and through generation of a crystallographic symmetry mate (Fig. 3B and Supplementary Fig. S2). The final model of the apo *Pa*DHDPR monomer included 264 amino acid residues and 296 water molecules, with an overall topology comprising eight α-helices and 11 β-strands. DynDom analysis of *Pa*DHDPR structures show distinct N- and C-terminal domains linked by two hinge regions (residues 129–133 and 233–235), with a 32.3° rotation angle and 2.1-Å translational movement^39^. The N-terminal domain (residues 1–127 and 235–264) responsible for cofactor binding comprised a parallel six-stranded β-sheet surrounded by six α-helices. The C-terminal domain (residues 128–234) responsible for substrate binding and tetramer formation included a five-stranded β-sheet and two α-helices. In the tetramer, each C-terminal domain formed a central 20-stranded β-barrel structure surrounded by eight α-helices. Each N-terminal domain was located outside of the tetrameric core structure formed by the four C-terminal domains. These structural features are similar to those reported for other DHDPR tetramers. A structural homology search using the DaliLite server (https://www.ebi.ac.uk/Tools/structure/dalilite/) revealed that DHDPR from various species had high Z scores, with the highest score observed for *Av*DHDPR (29.7), followed by NADH-bound *Mt*DHDPR (PDB: 1YL7; 28.8) and DHDPR from *C. glutamicum* (PDB: 5EER; 28.5)^25,40,41^.

**Figure 4:**
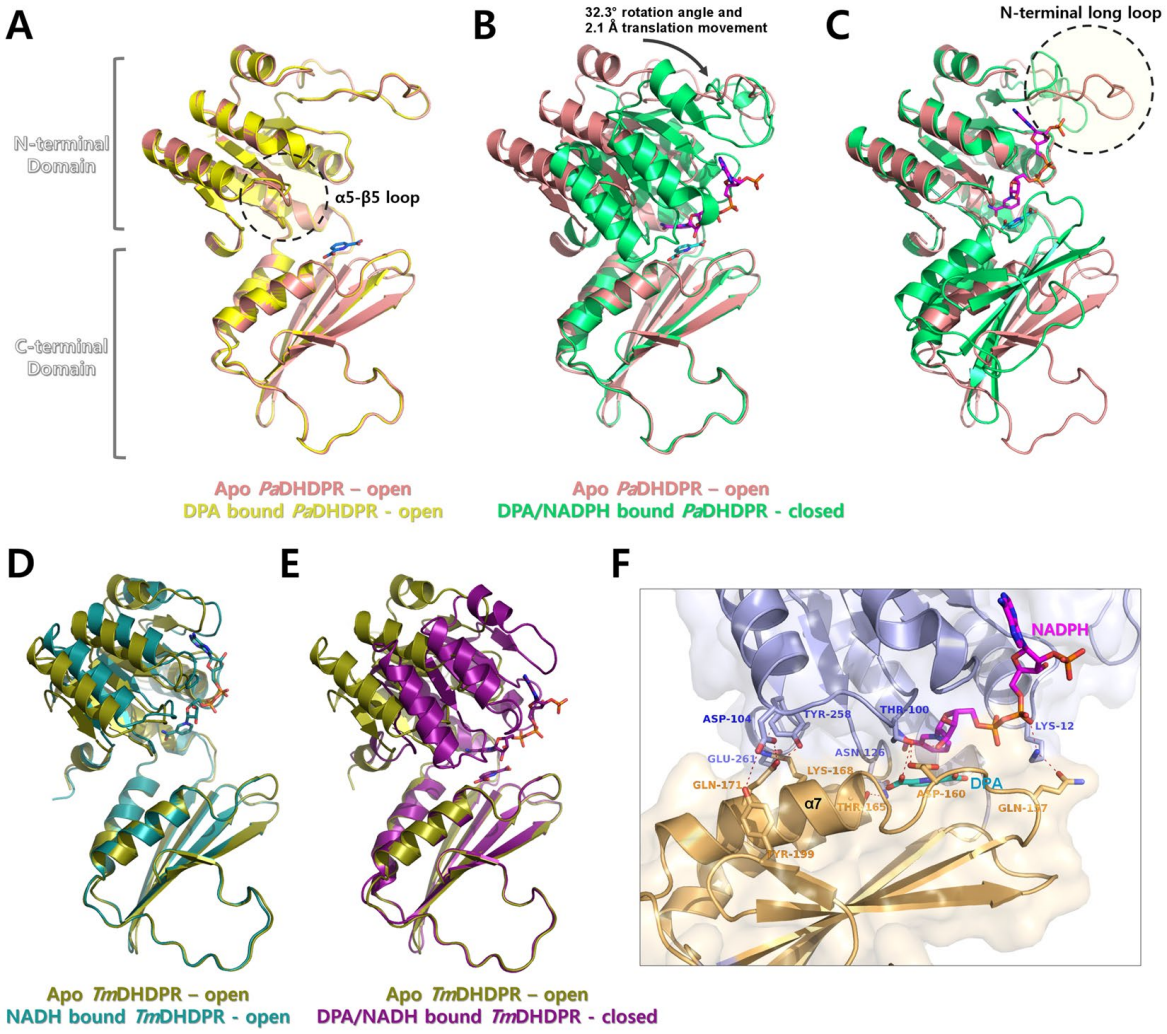
Domain movement and conformational change in *Pa*DHDPR by substrate and cofactor binding. (**A**) Structure superposition of apo (salmon) and DPA-bound (yellow) *Pa*DHDPR. Bound DPA is represented as a stick model. (**B**) Structural alignment of the C-terminal domains of apo (salmon) and NADPH/DPA-bound (lime green) *Pa*DHDPR showing rotation of the N-terminal domain. (**C**) Structural alignment of the N-terminal domains of apo (salmon) and NADPH/DPA-bound (lime green) *Pa*DHDPR showing conformational change in the NLL region. (**D**) Superimposition of the apo (PDB: 5TEK; deep olive) and NADH-bound (PDB: 1Y17; deep teal) forms of *Tm*DHDPR revealed only partial rotation, indicating that NADPH binding alone is insufficient to induce a fully closed state. (**E**) Superimposition of the apo and DPA/NADH-bound (PDB: 1C3V; deep purple) forms of *Tm*DHDPR revealed full domain closure. (**F**) The domain interface between N-terminal (light blue) and C-terminal (light orange) domains of the fully closed state of *Pa*DHDPR shows that an interaction network is formed by direct interactions between these domains, as well as interactions mediated by bound NADPH and DPA.

The crystal structure of DPA-bound *Pa*DHDPR was also obtained in the hexagonal space group *P*6_4_22 and solved by molecular replacement using the apo form as a search model. Electron density corresponding to DPA was observed in the substrate-binding site of the C-terminal domain. Structural superposition of apo and DPA-bound *Pa*DHDPR revealed that the two conformations were highly similar, with a root-mean-square deviation of 0.54 Å (over 244 Cα atoms) (Fig. 4A). This suggests that DPA alone cannot induce domain closure of *Pa*DHDPR. The structure of the ternary complex (NADPH/DPA-bound *Pa*DHDPR) was determined by molecular replacement using separate domain-structure models of apo *Pa*DHDPR. Clear electron density was observed in the putative N-terminal nucleotide-binding site that showed good correspondence with NADPH. The electron density of DPA was also observed adjacent to the NADPH-binding site. Comparison of the overall structures of apo and NADPH/DPA-bound *Pa*DHDPR showed a root-mean-square deviation of 3.6 Å (over 244 Cα atoms) due to domain motion initiated upon NADPH and DPA binding. By contrast, structural superposition of each domain of apo and NADPH/DPA-bound *Pa*DHDPR revealed relatively small differences, with root-mean-square deviations of 0.54 Å and 0.24 Å for the N- and C-terminal domains, respectively (over 125 and 84 Cα atoms, respectively) (Fig. 4B,C).

### Open and closed conformations of PaDHDPR

The N-terminal nucleotide-binding and C-terminal substrate-binding domains of *Pa*DHDPR are linked by two flexible hinge regions that mediate the opening and closing of the enzyme. The apo and DPA-bound forms of *Pa*DHDPR were in an open state (Fig. 4A), whereas the DPA/NADPH-bound enzyme was in a closed state (Fig. 4B). Similarly, the apo (PDB: 5TEK, unpublished) and nucleotide-bound (PDB: 1Y17) forms of *Tm*DHDPR were both in the open state (Fig. 4D)^40^, whereas the substrate- and nucleotide-bound ternary complexes existed in closed states (Fig. 4E). Therefore, DHDPR binding of both substrate and nucleotide is required to induce a conformational switch to the closed state for enzymatic activity. It should be noted that an NADH-bound *Mt*DHDPR structure showed a partially closed conformation, with the authors speculating that this unexpected closed structure might represent a snapshot of the equilibrium state between open and closed forms awaiting further substrate binding, and that subsequent substrate binding might induce the fully closed conformation^21^.

DPA binding in *Pa*DHDPR induced a small change in the α5–β5 loop region (Fig. 4A); however, it is unlikely that the change was due to DPA binding alone, as the distance between the loop and DPA is approximately 9 Å. In the closed state of *Pa*DHDPR, the interaction surface between the N- and C-terminal domains can be divided into two components: one for the interaction mediated by NADPH and DPA, and another for the interaction mediated by the α7 helix (Fig. 4F). Specifically, the NZ atom of Lys12 interacts with the NO1 atom of the adenine phosphate of NADPH, as well as with the OE1 atom of Gln157. The Thr100 residue interacts with NADPH and DPA and also forms a hydrogen bond with Asp160. The OG1 atom of Thr165 forms a hydrogen bond with the ND2 atom of Asn126, as well as with the O3 atom of DPA. Lys168 forms a salt bridge with Glu261, whereas Lys167 interacts with the main-chain O atom of Tyr258. Gln171 interacts with the side chain of Tyr258 and Asp104.

### The NADPH-binding site of PaDHDPR

**Figure 5:**
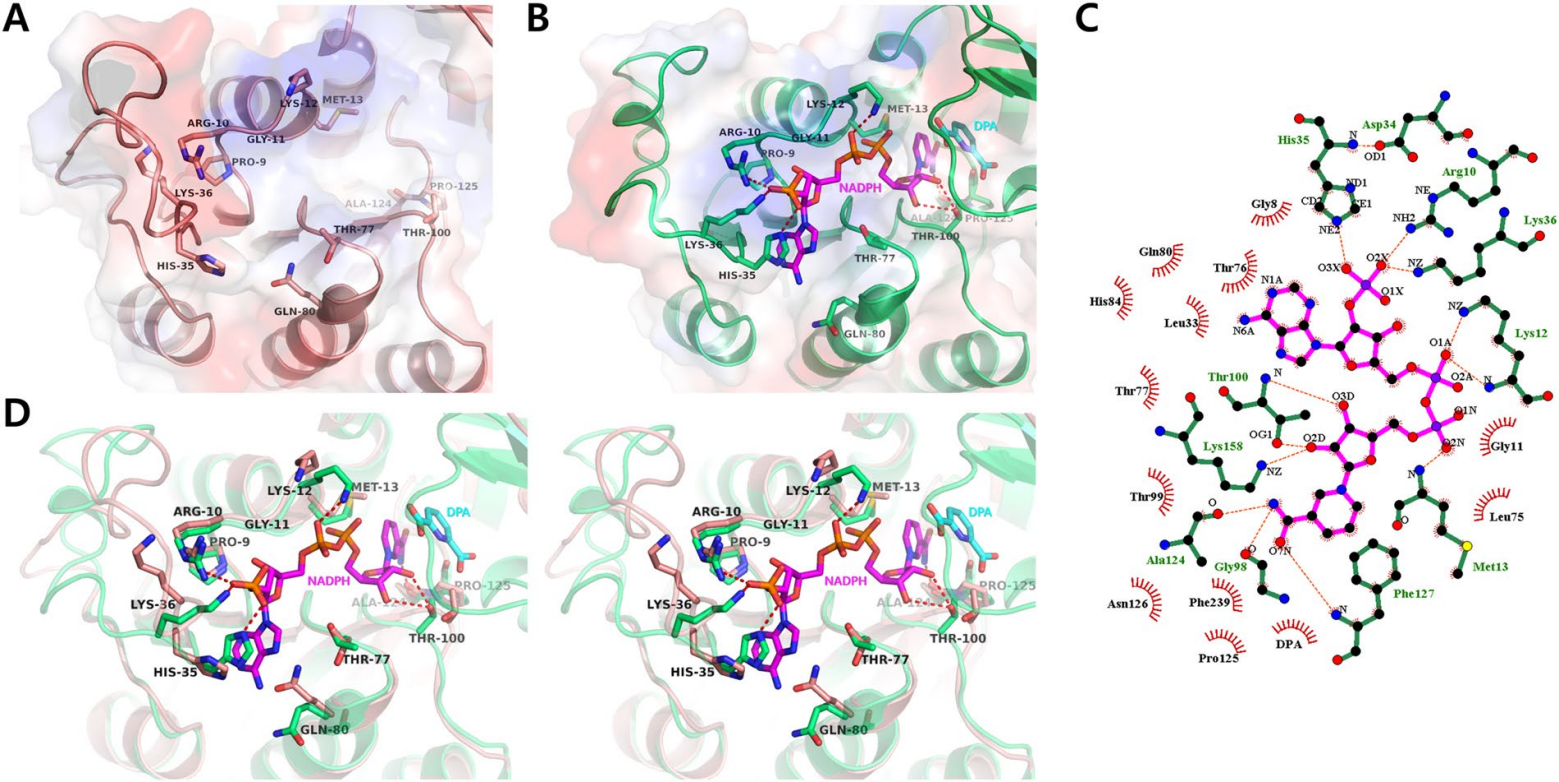
Structural comparison of the nucleotide-binding sites of apo and NADPH/DPA-bound *Pa*DHDPR. (**A**) Close-up view of the nucleotide-binding site of apo *Pa*DHDPR (salmon). Side chains of important residues around the nucleotide-binding site are indicated as sticks. (**B**) Close-up view of *Pa*DHDPR (lime green) and bound NADPH (magenta). Hydrogen bonds between NADPH and *Pa*DHDPR are represented as red dashed lines. (**C**) Ligplot diagram of NADPH binding in *Pa*DHDPR. Hydrogen bonds are illustrated by red dashed lines, and hydrophobic interactions are shown as curved red combs. (**D**) Stereo view of apo *Pa*DHDPR superposed with the NADPH/DPA-bound form of the enzyme. The NLL region undergoes conformational changes upon NADPH binding.

The N-terminal domain of *Pa*DHDPR contains the sequence G-x-x-G-x-x-G (Gly8, Pro9, Arg10, Gly11, Lys12, Met13, and Gly14), which is a conserved nucleotide-binding motif in DHDPRs. Additionally, the nucleotide-binding site of *Pa*DHDPR contains positive charges that match the negative charges of NADPH phosphates. Specifically, the O1A of adenine phosphate interacts with the NZ of Lys12, the C2 phosphate group of the adenine ribose interacts with the Arg10, His35, and Lys36 side chains, the O2X of adenine phosphate forms hydrogen bonds with the NH2 of Arg10 and the NZ of Lys36, the O3X of adenine phosphate forms hydrogen bonds with the NE2 atom of His35, the O2D and O3D of the nicotinamide ribose interact with OG1 of Thr100, and the main-chain oxygen atoms of Ala124 and Gly98 form hydrogen bonds with the N7N of the nicotinamide amide group (Fig. 5B,C). Structural superposition of the N-terminal domains of apo and NADPH/DPA-bound *Pa*DHDPR reveal conformational changes in the region including residues 34 through 59 and resulting from NADPH binding (Fig. 4B,C). In the apo form, this loop region shows a relatively high B-factor as compared with other regions. Additionally, the Gln80 side chain was flipped outward, and changes in Thr100 and Thr77 position were observed following NADPH binding (Fig. 5D).

### Comparison of NADPH-binding modes among various DHDPRs

Previous studies show that the N-terminal long-loop (NLL)-region residues are important for cofactor specificity in DHDPRs. *Ec*DHDPR can use both NADH and NADPH, although it exhibits a slight preference for NADH. In the NADH-bound *Ec*DHDPR structure, Glu38 in the NLL region interacts with the 2′ and 3′ hydroxyl groups of the NADH adenosyl ribose. On the other hand, Arg39 is involved in NADPH binding to *Ec*DHDPR through direct interaction with the adenine phosphate of NADPH. Therefore, these two residues in the NLL region might confer *Ec*DHDPR with dual specificity^26^. It is also possible that the Lys or Arg residues in the NLL region might play critical roles in NADPH preference in DHDPRs, such as *Tm*DHDPR and *Sa*DHDPR.

**Figure 6:**
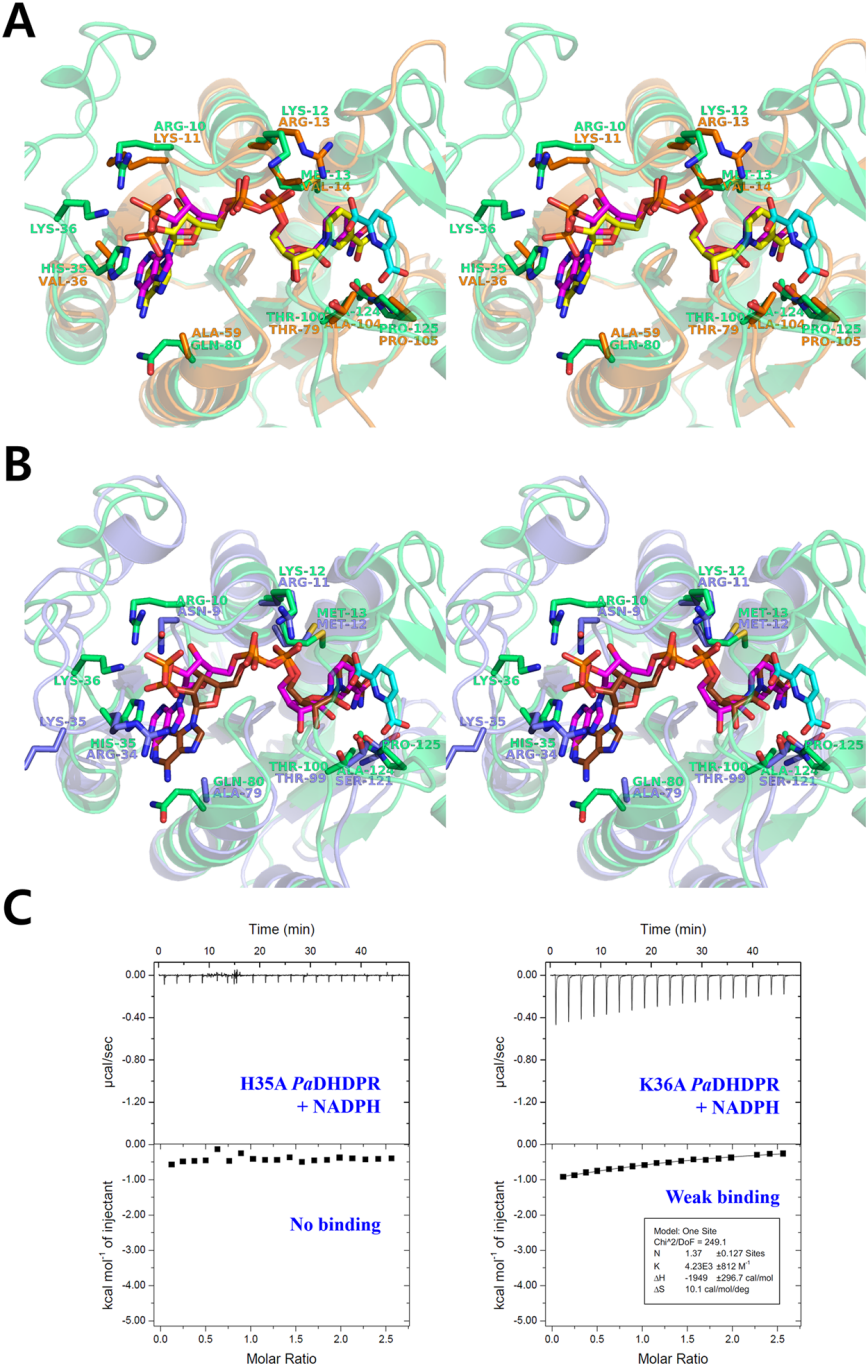
Structural comparisons of NADPH (magenta)-bound *Pa*DHDPR (lime green) with (**A**) NADPH (yellow)-bound *Cg*DHDPR (PDB: 5EES; orange) and (**B**) NADPH (brown)-bound *Bh*DHDPR (PDB: 3IJP S; slate). Each model is represented as a cartoon with sticks in stereo view. (**C**) Profile from ITC experiments using H35A (left panel) and K36A (right panel) mutant *Pa*DHDPR with NADPH showing significantly reduced binding affinity relative to that of wild-type *Pa*DHDPR.

Structural comparison of the N-terminal domains of NADPH/DPA-bound *Pa*DHDPR and NADPH-bound *C. glutamicum* (*Cg*)DHDPR (PDB 5EES; sequence identity: 44%) revealed two key differences in the NADPH-binding mode (Fig. 6A)^25^. *Cg*DHDPR lacks residues equivalent to His35 and Lys36 in *Pa*DHDPR due to a very short NLL region (Fig. 3C). Additionally, Lys11, which is equivalent to Arg10 in *Pa*DHDPR, forms a salt bridge with Glu33 instead of interacting with the adenine phosphate group of NADPH. These structural features explain the promiscuity of *Cg*DHDPR in regard to cofactor preference between NADH and NADPH.

*B*. *henselae* (*Bh*)DHDPR (PDB: 3IJP; sequence identity: 27%) also contains an NLL region, but its interaction with the adenine phosphate of NADPH differs significantly from that of *Pa*DHDPR (Fig. 6B) due to differences in local structure and amino acid sequence^24^. Arg34 of *Bh*DHDPR, which is the equivalent to His35 in *Pa*DHDPR, forms a charged interaction with the adenine phosphate of NADPH. However, Lys35 (Lys36 in *Pa*DHDPR) in the NLL region is exposed to solvent in the absence of any specific interaction with bound NADPH. Moreover, Asn9 in *Bh*DHDPR and located in the β1–α1 loop interacts with the ribose AO3 of NADPH. Based on these observations, *Bh*DHDPR might harbour a weak cofactor preference for NADPH over NADH.

Our comparative structural analyses demonstrate that *Pa*DHDPR has more specific interactions with the adenine phosphate of NADPH. The ITC analysis confirmed that H35A mutation induced complete loss of binding affinity and K36A mutants had approximately 6.4 folds reduced affinity (*Kd* value of 236 µM) for NADPH compared with that of wild-type *Pa*DHDPR (*Kd* value of 37 µM) (Fig. 6C). Therefore, our findings suggested that residues located in the NLL region of *Pa*DHDPR engage in specific interactions with NADPH. We speculated that DHDPR preference for a given form of NAD(P)H is determined by the combination of various residues in this region. Notably, our sequence-alignment results showed that the residues therein vary considerably across species (Fig. 3C), with *Tm*DHDPR, which has a strong preference for NADPH, having a very short NLL region^22^. Additional biochemical and structural data are needed in order to determine the cofactor preference of DHDPRs based solely on sequence information.

### The substrate-binding site of PaDHDPR

**Figure 7:**
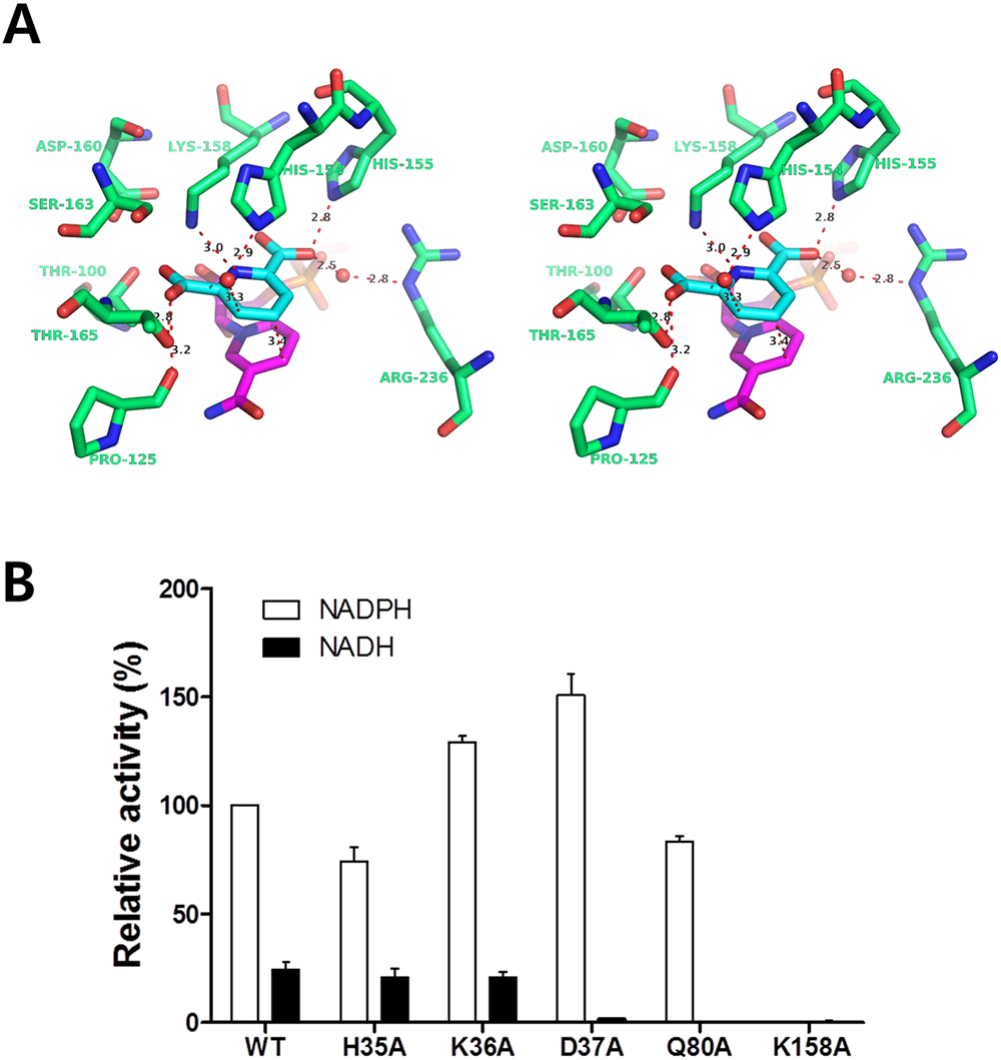
DPA-binding mode and activity assay of *Pa*DHDPR using several mutants. (**A**) Stereo view of the DPA-binding mode of *Pa*DHDPR. DPA (cyan) and its interacting residues (green) in *Pa*DHDPR are shown as a stick model. (**B**) Comparison of relative activities between *Pa*DHDPR wild-type and several mutants. Assays were performed with NADH or NADPH as a cofactor under standard assay conditions. The relative activity of wild-type *Pa*DHDPR with NADPH was set to 100%. Experiments were performed in triplicate, and the error bar indicates the standard deviation.

To investigate the substrate-binding mechanism of *Pa*DHDPR, we determined the structures of DPA- and DPA/NADPH-bound forms of the enzyme (Fig. 7A). DPA is a competitive inhibitor of DHDPR and has a structure similar to DHDP, a *Pa*DHDPR substrate. In the ternary complex, the aromatic ring of DPA faces the nicotinamide ring of NADPH and separated by a distance of 3.4 Å to 4 Å. We also observed that the carboxyl group O3 of DPA interacts with the OG1 of Thr165 and the main-chain O of Pro125, and that the carboxyl group O1 on the opposite side of the of DPA molecule interacts with the NE2 of His155. The NE of Arg236 interacts with the O1 of DPA, with this interaction mediated by a water molecule. Lys158 is involved in substrate polarization and enamine stabilization^26^. Here, we found here that Lys158 directly interacts with the N1 of DPA, with its importance for enzymatic activity confirmed by enzyme inactivity following introduction of a K158A mutation (Fig. 7B).

## Conclusion

In this study, we performed biochemical and structural characterization of *Pa*DHDPR and provided the first report on a DHDPR from psychrophilic bacteria. Although *Pa*DHDPR showed maximal activity at 40 °C, it retained >40% of its activity at 10 °C. Comparative structural analyses suggested that transition between the open and closed conformations in DHDPRs is determined by the binding of both substrate and nucleotide, given that only the DPA/NADPH-bound form of *Pa*DHDPR showed a closed conformation. Moreover, NADPH binding induced a dramatic conformational change in the NLL region of *Pa*DHDPR, with His35 and Lys36 in this region interacting with the adenine phosphate of NADPH and critical for nucleotide binding. The activity of NADH-bound *Pa*DHDPR was ~20% to 30% lower than that of the NADPH-bound enzyme, indicating that *Pa*DHDPR prefers NADPH as a cofactor, but can still use NADH despite a very low affinity. Interestingly, the K36A mutant exhibited reduced NADPH-binding affinity according to ITC results, but exhibited slightly increased activity relative to that of the wild-type variant according to activity assays. This might be a consequence of the DHDP substrate promoting NADPH binding in the K36A mutant. In our DPA/NADPH-bound complex structure, the aromatic ring of DPA forms π-π stacking interaction with the nicotinamide ring of NADPH (Fig. 7A). It is also possible that the K36A mutant has a more flexible NLL region that might allow a more rapid transition between the open and closed conformations during enzyme activity. Similarly, Asp37 located in the NLL region is important for loop stabilization, and the D37A mutant also showed a slight increase in enzyme activity as compared with that of wild-type *Pa*DHDPR (Fig. 7B). Therefore, we speculated that destabilization of the NLL structure might result in elevated *Pa*DHDPR activity (Supplementary Fig. S3). These findings provide a basis for the development of drugs against spore-forming bacteria that act by targeting the lysine-synthesis pathway.

## Methods

### PaDHDPR cloning, expression, and purification

The gene encoding DHDPR (residues 1–264) was amplified by polymerase chain reaction (PCR) from the genomic DNA of *Paenisporosarcina* sp. TG-14 using PrimeSTAR HS DNA polymerase (Takara Bio, Otsu, Japan) and the primers 5′-CGATAACATATGATGATTCGAGTTGCGATTG-3′ (forward) and 5′-CGATAACTCGAGTTATAAAATATTTTCTAG-3′ (reverse). The DNA fragment was cloned into the *Nde*I and *Xho*I sites of the pET-28a vector (Novagen, Madison, WI, USA). The construct, which introduced a cleavable N-terminal His tag, was transformed into *E. coli* BL21(DE3) competent cells for protein expression. The cells were cultured at 37 °C in 4 L of Luria-Bertani (LB) medium containing 50 µg ml^−1^ kanamycin until the optical density at 600 nm (OD_600_) was ~0.6. *Pa*DHDPR expression was induced overnight at 25 °C by 0.4 mM isopropyl-1-thio-β-d-galactopyranoside (IPTG). The cells were harvested by centrifugation and lysed by sonication in lysis buffer comprising 50 mM sodium phosphate, 300 mM NaCl, and 5 mM imidazole (pH 8.0) with 0.2 mg ml^−1^ lysozyme. The debris was removed by centrifugation at 16,000 rpm for 1 h at 4 °C. The supernatant was applied to an Ni-nitroloacetic acid affinity column (Qiagen, Hilden, Germany) pre-equilibrated with lysis buffer and then washed with wash buffer comprising 50 mM sodium phosphate, 300 mM NaCl, and 20 mM imidazole (pH 8.0). The target protein was eluted with elution buffer comprising 50 mM sodium phosphate, 300 mM NaCl, and 300 mM imidazole (pH 8.0) and concentrated using Amicon ultracentrifugal filters (Ultracel-3K; Millipore, Darmstadt, Germany). The His tag of the purified protein was removed by thrombin treatment, and the protein was applied to a Superdex 200 column (GE Healthcare, Piscataway, NJ, USA) pre-equilibrated with buffer comprising 50 mM Tris-HCl (pH 8.0) and 150 mM NaCl. Peak fractions containing *Pa*DHDPR were collected and concentrated.

Selenomethionine (SeMet)-labelled *Pa*DHDPR was expressed in *E. coli* BL21(DE3) competent cells. The transformed cells were grown at 37 °C in 2 l SeMet medium (Molecular Dimensions, Maumee, OH, USA) containing 50 µg ml^−1^ kanamycin until the OD_600_ was ~0.6. Protein expression was induced overnight at 25 °C with 0.4 mM IPTG. SeMet-labeled *Pa*DHDPR was purified in the same manner as native *Pa*DHDPR.

### Enzymatic activity assay

DHDPR activity was evaluated at 25 °C via a coupled reaction with DHDPS (*dapA*) to form the substrate, as previously described^42^. *Pa*DHDPS was cloned, expressed, and purified in the same manner as *Pa*DHDPR. The standard reaction mixture contained 100 mM HEPES (pH 7.5), 0.1 mM NAD(P)H, 1 mM sodium pyruvate, 0.1 mM ASA, an excess of *Pa*DHDPS (25 μg), and *Pa*DHDPR. After 1 min of incubation with *Pa*DHDPS for complete conversion of ASA to DHDP, the reaction was initiated by adding *Pa*DHDPR. DHDPR activity was measured by monitoring the decrease in absorbance of NAD(P)H at 340 nm (molecular extinction coefficient = 6.22 mM^−1^ cm^−1^) with a spectrophotometer (UV-1800; Shimadzu, Kyoto, Japan). Kinetic parameters were determined by varying the concentration of NAD(P)H or ASA. When DHDPR was assayed with known concentrations of ASA, the concentration of NADP or NADPH varied. The kinetic parameters for ASA were determined by varying ASA concentration and maintaining a fixed concentration of NADPH. Enzyme *K*_m_ and *k*_cat_ were estimated by fitting the initial rate data to the Michaelis-Menten equation by nonlinear regression using Prism software (v.5; GraphPad, Inc., San Diego, CA, USA).

### Optimal pH and temperature

The optimal pH was determined by measuring enzymatic activity at various pH values ranging from 5.0 to 9.0 under standard assay conditions. Various buffers were used, including 100 mM sodium acetate (pH 5.0–6.5), 100 mM HEPES (pH 6.5–8.5), and 100 mM Tris-HCl (pH 8.0–9.0). The optimal temperature was determined by performing the assay at temperatures ranging from 5 °C to 60 °C. Maximal enzymatic activity was set as 100% relative activity.

### ITC

ITC experiments were performed using a MicroCal Auto-iTC200 instrument (GE Healthcare) at the Korea Basic Science Institute (Daejeon, Korea). Protein and ligand solutions were prepared in buffer comprising 50 mM Tris-HCl and 150 mM NaCl (pH 8.0), and all titrations were performed at 25 °C. The sample cell containing 80 μM *Pa*DHDPR was titrated with 1 mM NADH or NADPH in the syringe. Data were fitted to a one-site binding model using MicroCal ORIGIN software, which was provided with the instrument.

### Crystallization and data collection

Purified native *Pa*DHDPR protein was concentrated to 215.95 mg ml^−1^. Initial crystallization screening was performed at 293 K in 96-well crystallization plates (Emerald Bio, Bainbridge Island, WA, USA) using a crystallization robotic nanoliter sitting-drop system (Mosquito; TTP Labtech, Cambridge, MA, USA) and a commercially available screening kit. The drops contained 300 nl each of protein and reservoir solutions and were equilibrated against 80 μl reservoir solution. Crystals of *Pa*DHDPR were grown under conditions of 30% (v/v) polyethylene glycol (PEG)400, 100 mM Tris-HCl (pH 8.5), and 200 mM MgCl_2_ (Wizard Classic #C1). Crystals of SeMet-labeled *Pa*DHDPR were obtained in the same manner under the same conditions. Co-crystals of DPA-bound *Pa*DHDPR with 10 mM DPA were obtained from the condition of 30% (v/v) PEG 400, 100 mM HEPES:NaOH (pH 7.5), and 200 mM MgCl_2_ (MCSG2 #H8). To determine the structure of the NADPH/DPA-bound complex, 10 mM NADPH and 10 mM DPA were added to *Pa*DHDPR protein solution for co-crystallization. Crystals of NADPH/DPA-bound *Pa*DHDPR were obtained under conditions of 0.2 M ammonium sulphate, 0.1 M Tris-HCl (pH 8.5), and 25% (w/v) PEG 3350 (MCSG1 #G8). Crystals were directly harvested from the initial screening plate and transferred to cryoprotectant Paratone-N oil (Hampton Research, Aliso Viejo, CA, USA) and flash-frozen with a liquid-nitrogen gas stream. X-ray diffraction data were collected using a BL-5C beam line at the Pohang Accelerator Laboratory (Pohang, Korea). X-ray diffraction data for SeMet-labelled *Pa*DHDPR included 360 images collected at a wavelength of 0.9790 Å. Data for native, DPA-bound, and NADPH/DPA-bound *Pa*DHDPR included 120, 360, and 360 images, respectively. Images were collected at 1° oscillation with an exposure time of 1 s per image. Diffraction data were indexed, processed, and scaled using the HKL-2000 program^43^. X-ray diffraction data statistics are presented in Table 1.

**Table 1:** X-ray diffraction data and refinement statistics.

**Dataset**	**SeMet-labelled** ***Pa*** **DHDPR**	**Native apo** ***Pa*** **DHDPR**	**DPA-bound** ***Pa*** **DHDPR**	**NADPH/DPA-bound** ***Pa*** **DHDPR**
X-ray source	PAL 5C beam line	PAL 5C beam line	PAL 5C beam line	PAL 5C beam line
Space group	*P*6_4_22	*P*6_4_22	*P*6_4_22	*P*4_2_2_1_2
Unit cell parameters (Å, °)	a = b = 106.466, c = 102.161 α = β = 90, γ = 120	a = b = 106.229, c = 101.999 α = β = 90, γ = 120	a = b = 105.855, c = 101.472 α = β = 90, γ = 120	a = b = 79.942, c = 83.835 α = β = γ = 90
Wavelength (Å)	0.9790	0.9795	0.9795	0.9796
Resolution (Å)	50.00–2.00 (2.03–2.00)	50.00–1.80 (1.83–1.80)	50.00–1.80 (1.83–1.80)	50.00–2.10 (2.14–2.10)
Total reflections	983052	350105	1342083	217098
Unique reflections	23579 (1146)	31125 (1554)	31600 (1544)	16378 (781)
Average I/σ (I)	122.0 (58.5)	31.8 (6.6)	89.2 (12.75)	40.9 (4.7)
*R*_merge_^a^	0.157 (0.439)	0.064 (0.122)	0.073 (0.460)	0.080 (0.494)
Redundancy	41.7 (42.3)	11.3 (13.0)	43.5 (42.5)	13.3 (12.6)
Completeness (%)	99.9 (100.0)	97.2 (99.9)	100.0 (100.0)	99.3 (98.5)
**Refinement**				
Resolution range (Å)		47.11–1.80 (1.85–1.80)	46.93–1.80 (1.85–1.80)	50.01–2.10 (2.15–2.10)
No. of reflections of working set		29463 (2165)	29965 (2143)	15536 (1092)
No. of reflections of test set		1596 (111)	1598 (122)	783 (55)
No. of amino acid residues		264	264	265
No. of water molecules		293	293	125
*R*_cryst_^b^		0.167 (0.164)	0.160 (0.169)	0.187 (0.288)
*R*_free_^c^		0.207 (0.243)	0.192 (0.229)	0.257 (0.334)
R.m.s. bond length (Å)		0.022	0.023	0.014
R.m.s. bond length (°)		2.136	2.104	1.807
Average B value (Å2) (protein)		22.579	20.378	39.371
Average B value (Å2) (solvent)		38.585	36.022	41.599

^a^*R*_merge_ = Σ | <I> − I |/Σ <I>.

^b^*R*_cryst_ = Σ | |Fo| − |Fc| |/Σ |Fo|.

^c^*R*_free_ calculated with 5% of all reflections excluded from refinement stages using high-resolution data.

Values in parentheses refer to the highest resolution shells.

### Structure determination and refinement

The *Pa*DHDPR structure was solved by SAD^38^. The 2.0-Å data of Se-Met-labelled *Pa*DHDPR was collected at the energy of the Se edge (12.6645 keV) obtained from X-ray energy scans. The *AutoSol* program from *PHENIX* generated a partial structure model with *R*_work_ and *R*_free_ values of 27.7% and 29.9%, respectively^44,45^. The structure of native *Pa*DHDPR was solved by molecular replacement with *MOLREP* from the *CCP4* suite using the partial model of SeMet-labelled *Pa*DHDPR^46,47^. The model was improved through iterative cycles of manual model building with *Coot* and refinement with *REFMAC* in the *CCP4* suite^48,49^. The final model had *R*_work_ and *R*_free_ values of 16.9% and 20.7%, respectively. Molecular replacement for DPA- and NADPH/DPA-bound *Pa*DHDPR was performed using apo *Pa*DHDPR and its individual domains. Rebuilding and refinement were performed in the same manner as for apo *Pa*DHDPR. The final model of DPA-bound *Pa*DHDPR had *R*_work_ and *R*_free_ values of 16.0% and 19.5%, respectively, whereas the final model of NADPH/DPA-bound *Pa*DHDPR had *R*_work_ and *R*_free_ values of 18.7% and 25.7%, respectively. The quality of the final structure models was verified using *MolProbity*^50^. Detailed refinement statistics are listed in Table 1. Structure factors and coordinates of apo and DPA- and NADPH/DPA-bound *Pa*DHDPR have been deposited in the PDB with codes 5Z2D, 5Z2E, and 5Z2F, respectively.

## Electronic supplementary material

Supplementary information

## References

[1] Grossman AD (1995). Genetic networks controlling the initiation of sporulation and the development of genetic competence in *Bacillus subtilis*. Annu. Rev. Genet..

[2] Errington J (2003). Regulation of endospore formation in *Bacillus subtilis*. Nat. Rev. Microbiol..

[3] Burns DA, Minton NP (2011). Sporulation studies in *Clostridium difficile*. J. Microbiol. Methods.

[4] Molle V (2003). The Spo0A regulon of *Bacillus subtilis*. Mol. Microbiol..

[5] Eichenberger P (2004). The program of gene transcription for a single differentiating cell type during sporulation in *Bacillus subtilis*. PLoS Biol..

[6] Roszak D, Colwell R (1987). Survival strategies of bacteria in the natural environment. Microbiol. Rev..

[7] Black S, Wright NG (1955). Aspartic β-semialdehyde dehydrogenase and aspartic β-semialdehyde. J. Biol. Chem..

[8] Yugari Y, Gilvarg C (1965). The condensation step in diaminopimelate synthesis. J. Biol. Chem..

[9] Laber B, Gomis-Rüth F, Romao M, Huber R (1992). *Escherichia coli* dihydrodipicolinate synthase. Identification of the active site and crystallization. Biochem. J..

[10] Fukuda A, Gilvarg C (1968). The relationship of dipicolinate and lysine biosynthesis in Bacillus megaterium. J. Biol. Chem..

[11] Slieman TA, Nicholson WL (2001). Role of dipicolinic acid in survival of *Bacillus subtilis* spores exposed to artificial and solar UV radiation. Appl. Environ. Microbiol..

[12] Setlow B, Atluri S, Kitchel R, Koziol-Dube K, Setlow P (2006). Role of dipicolinic acid in resistance and stability of spores of *Bacillus subtilis* with or without DNA-protective α/β-type small acid-soluble proteins. J. Bacteriol..

[13] Jamroskovic J (2016). Variability in DPA and calcium content in the spores of *Clostridium* species. Front. Microbiol..

[14] Liu Y, Xie S, Yu J (2016). Genome-wide analysis of the lysine biosynthesis pathway network during maize seed development. PloS One.

[15] Guinand M, Michel G, Tipper D (1974). Appearance of a γ-d-glutamyl-(l) meso-diaminopimelate peptidoglycan hydrolase during sporulation in *Bacillus sphaericus*. J. Bacteriol..

[16] Pavelka M, Jacobs WR (1996). Biosynthesis of diaminopimelate, the precursor of lysine and a component of peptidoglycan, is an essential function of *Mycobacterium smegmatis*. J. Bacteriol..

[17] Galili G (1995). Regulation of lysine and threonine synthesis. Plant Cell.

[18] Paiva AM (2001). Inhibitors of dihydrodipicolinate reductase, a key enzyme of the diaminopimelate pathway of *Mycobacterium tuberculosis*. Biochim. Biophys. Acta.

[19] Farkas W, Gilvarg C (1965). The reduction step in diaminopimelic acid biosynthesis. J. Biol. Chem..

[20] Scapin G, Blanchard JS, Sacchettini JC (1995). Three-dimensional structure of *Escherichia coli* dihydrodipicolinate reductase. Biochemistry.

[21] Cirilli M, Zheng R, Scapin G, Blanchard JS (2003). The three-dimensional structures of the *Mycobacterium tuberculosis* dihydrodipicolinate reductase– NADH– 2, 6-PDC and –NADPH– 2, 6-PDC complexes. Structural and mutagenic analysis of relaxed nucleotide specificity. Biochemistry.

[22] Yang Z (2003). Aspartate dehydrogenase, a novel enzyme identified from structural and functional studies of TM1643. J. Biol. Chem..

[23] Girish TS, Navratna V, Gopal B (2011). Structure and nucleotide specificity of *Staphylococcus aureus* dihydrodipicolinate reductase (DapB). FEBS Lett..

[24] Cala AR (2016). The crystal structure of dihydrodipicolinate reductase from the human-pathogenic bacterium *Bartonella henselae* strain Houston-1 at 2.3 Å resolution. Acta Crystallogr. F Struct. Biol. Comm..

[25] Sagong H-Y, Kim K-J (2016). Structural insight into dihydrodipicolinate reductase from *Corybebacterium glutamicum* for lysine biosynthesis. J. Microbiol. Biotechnol..

[26] Scapin G, Reddy SG, Zheng R, Blanchard JS (1997). Three-dimensional structure of *Escherichia coli* dihydrodipicolinate reductase in complex with NADH and the inhibitor 2, 6-pyridinedicarboxylate. Biochemistry.

[27] Pearce FG, Sprissler C, Gerrard JA (2008). Characterization of dihydrodipicolinate reductase from *Thermotoga maritima* reveals evolution of substrate binding kinetics. J. Biochem..

[28] Dommaraju SR (2011). Catalytic mechanism and cofactor preference of dihydrodipicolinate reductase from methicillin-resistant *Staphylococcus aureus*. Arch. Biochem. Biophys..

[29] Koh HY (2012). Draft genome sequence of *Paenisporosarcina* sp. strain TG-14, a psychrophilic bacterium isolated from sediment-laden stratified basal ice from Taylor Glacier, McMurdo Dry Valleys, Antarctica. J. Bacteriol..

[30] Price PB (2000). A habitat for psychrophiles in deep Antarctic ice. Proc. Natl. Acad. Sci. USA.

[31] Christner BC (2000). Recovery and identification of viable bacteria immured in glacial ice. Icarus.

[32] Mueller DR, Vincent WF, Bonilla S, Laurion I (2005). Extremotrophs, extremophiles, and broadband pigmentation strategies in a high arctic ice shelf ecosystem. FEMS Microbiol. Ecol..

[33] Kimura K, Goto T (1977). Dihydrodipicolinate reductases from *Bacillus cereus* and *Bacillus megaterium*. J. Biochem..

[34] Park S-H (2017). Crystal structure and functional characterization of an isoaspartyl dipeptidase (CpsIadA) from *Colwellia psychrerythraea* strain 34H. PloS One.

[35] Lee CW (2017). Crystal structure and functional characterization of an esterase (EaEST) from *Exiguobacterium antarcticum*. PloS One.

[36] Tyagi VV, Henke RR, Farkas WR (1983). Partial purification and characterization of dihydrodipicolinic acid reductase from maize. Plant Physiol..

[37] Griffin MD (2012). Characterisation of the first enzymes committed to lysine biosynthesis in *Arabidopsis thaliana*. PloS One.

[38] Hendrickson WA, Horton JR, LeMaster DM (1990). Selenomethionyl proteins produced for analysis by multiwavelength anomalous diffraction (MAD): a vehicle for direct determination of three‐dimensional structure. EMBO J..

[39] Hayward S, Lee RA (2002). Improvements in the analysis of domain motions in proteins from conformational change: DynDom version 1.50. J. Mol. Graph. Model..

[40] Janowski R, Kefala G, Weiss MS (2010). The structure of dihydrodipicolinate reductase (DapB) from *Mycobacterium tuberculosis* in three crystal forms. Acta Crystallogr. D Biol. Crystallogr..

[41] Holm L, Sander C (1995). Dali: a network tool for protein structure comparison. Trends Biochem. Sci..

[42] Coulter CV, Gerrard JA, Kraunsoe JAE, Pratt AJ (1999). *Escherichia coli* dihydrodipicolinate synthase and dihydrodipicolinate reductase: kinetic and inhibition studies of two putative herbicide targets. Pest Manag. Sci..

[43] Otwinowski Z, Minor W (1997). Processing of X-ray diffraction data collected in oscillation mode. Methods Enzymol..

[44] Terwilliger TC (2009). Decision-making in structure solution using Bayesian estimates of map quality: the PHENIX AutoSol wizard. Acta Crystallogr. D Biol. Crystallogr..

[45] Adams PD (2010). PHENIX: a comprehensive Python-based system for macromolecular structure solution. Acta Crystallogr. D Biol. Crystallogr..

[46] Vagin A, Teplyakov A (2010). Molecular replacement with MOLREP. Acta Crystallogr. D Biol. Crystallogr..

[47] Winn MD (2011). Overview of the CCP4 suite and current developments. Acta Crystallogr. D Biol. Crystallogr..

[48] Emsley P, Cowtan K (2004). Coot: model-building tools for molecular graphics. Acta Crystallogr. D Biol. Crystallogr..

[49] Murshudov GN (2011). REFMAC5 for the refinement of macromolecular crystal structures. Acta Crystallogr. D Biol. Crystallogr..

[50] Chen VB (2010). MolProbity: all-atom structure validation for macromolecular crystallography. Acta Crystallogr. D Biol. Crystallogr..

