# Supplementary material for: Crystal structure of dihydrodipicolinate reductase (PaDHDPR) from Paenisporosarcina sp. TG-14: structural basis for NADPH preference as a cofactor
Source: Sci Rep. 2018 May 21;8:7936. doi: 10.1038/s41598-018-26291-x (PMC5962572; doi:10.1038/s41598-018-26291-x)
Supplement: Supplementary file 1 — Supplementary information [file 41598_2018_26291_MOESM1_ESM.pdf]

Crystal structure of dihydrodipicolinate reductase (*Pa*DHDPR) from *Paenisporosarcina* sp. TG-14: structural basis for NADPH preference as a cofactor

Chang Woo Lee, Sun-Ha Park, Sung Gu Lee, Hyun Ho Park, Hak Jun Kim, HaJeung Park, Hyun Park, and Jun Hyuck Lee

Supplementary Information

Supplementary Table S1. Kinetic parameters of *Pa*DHDPR.

	**NADPH (constant ASA)**	**NADH (constant ASA)**	**ASA (constant NADPH)**
K_m (μM)	22 ± 1.4	43 ± 8.9	12 ± 1.6
k_{cat} (s^{-1})	55 ± 1.2	7.1 ± 0.1	52 ± 1.1
k_{cat}/K_m (s^{-1}/μM)	2.5 ± 0.2	0.17 ± 0.04	4.4 ± 0.6

Data are presented as the mean ± standard deviation of three independent experiments.
ASA, L-aspartate-semialdehyde; NADPH, nicotinamide adenine dinucleotide phosphate.

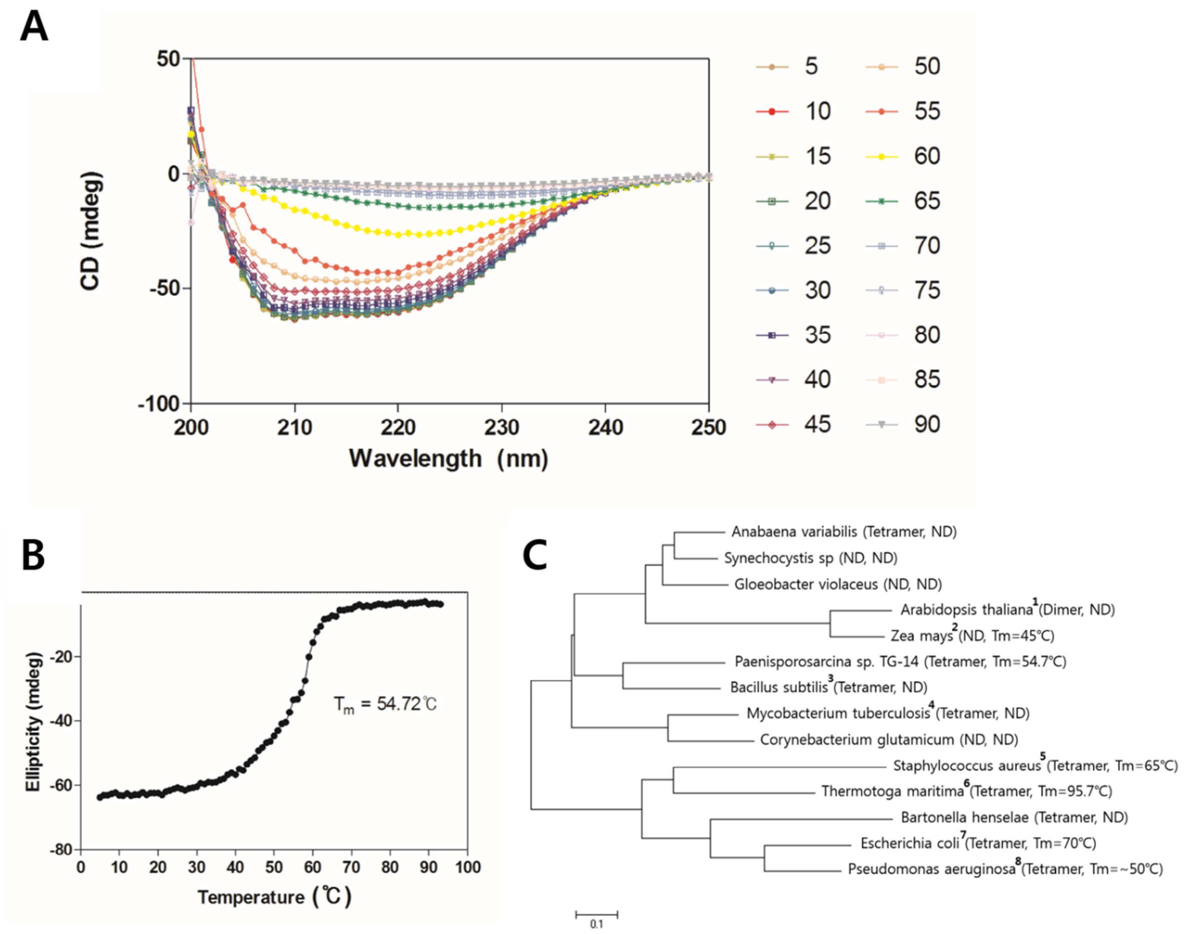

Supplementary Figure. S1. Thermal stability of *Pa*DHDPR monitored by circular dichroism spectroscopy. (a) Typical wavelength scan of *Pa*DHDPR was performed at a given temperature after a 1-min equilibration. (b) Thermal denaturation of *Pa*DHDPR was measured using circular dichroism (Chirascan CD spectropolarimeter; Applied Photophysics, Surrey, UK). *Pa*DHDPR protein solution (1 mg ml^{-1}) in 150 mM NaCl and 20 mM Tris-HCl (pH 8.0) was loaded onto a 0.1-cm path-length cuvette. Changes in ellipticity were monitored at a wavelength of 210 nm by heating the sample to between 5°C and 95°C at intervals of 1°C. The denaturation temperature (T_m) was defined as 54.72C°. (c) The phylogenetic tree based on a multiple sequence alignment of *Pa*DHDPR was drawn using ClustalX2 and MEGA4. The neighbouring-joining method and the bootstrap consensus tree were inferred. The oligomeric state and the melting temperature of DHDPR are given in parentheses.

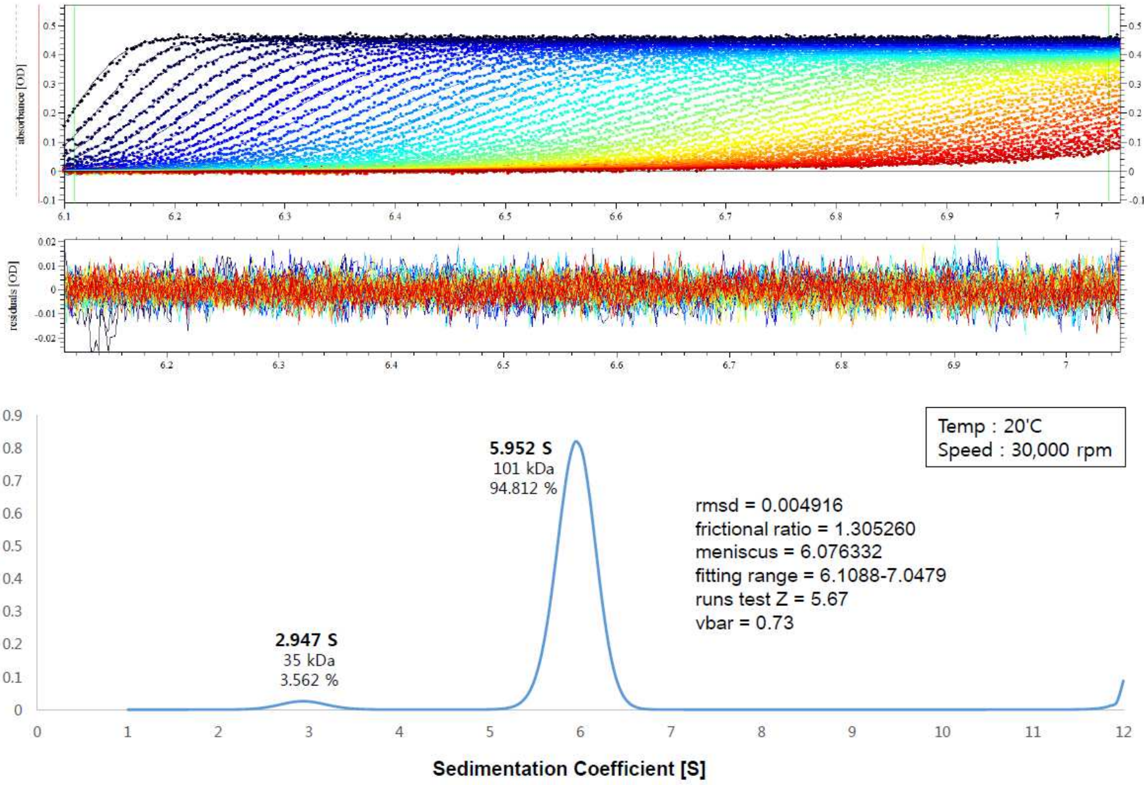

Supplementary Figure. S2. *Pa*DHDPR analytical ultracentrifugation (AUC). AUC experiments were performed using an XL-A analytical ultracentrifuge (Beckman Coulter, Brea, CA, USA). *Pa*DHDPR protein was diluted in buffer comprising 20 mM Tris-HCl (pH 8.0) and 150 mM NaCl to a final concentration of 0.5 mg/ml. Protein solution and buffer were loaded into the sample and reference sectors of the dual-sector epon centrepiece, respectively. Centrifugation was performed at 45,000 rpm, and sedimentation profile was monitored at 280 nm. Sedimentation-velocity data were analysed with the program SEDFIT[9,10]. AUC experiments on *Pa*DHDPR (residues 1–264; calculated molecular weight: 29 kDa for the polypeptide chain) yielded a mass of 101 kDa (sedimentation coefficient: 5.952 S; frictional ratio: 1.305), indicating that *Pa*DHDPR exists as a tetramer in solution.

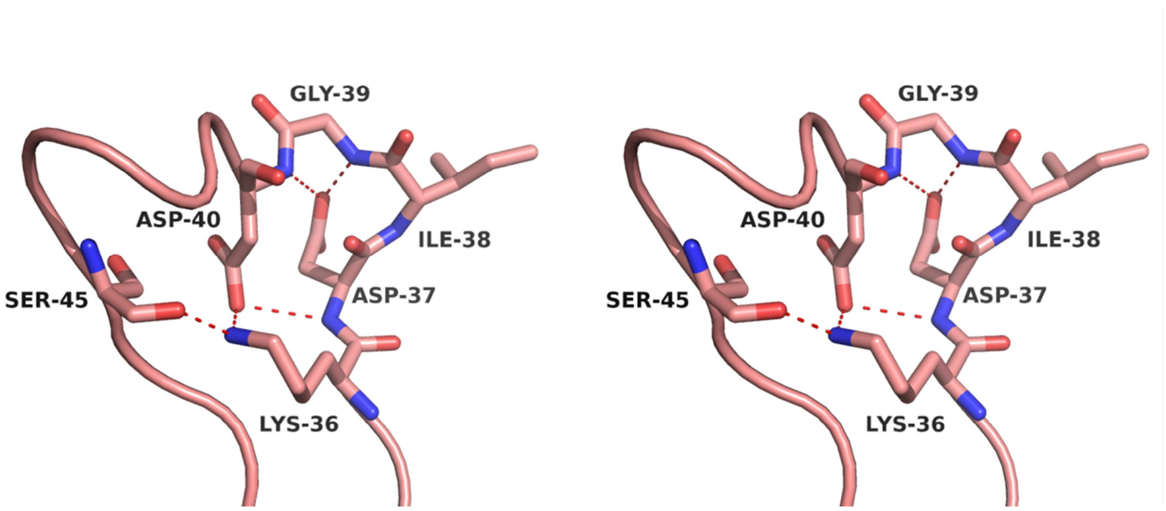

Supplementary Figure. S3. Stereo view of internal interactions in the N-terminal long loop (NLL) region. In the apo state, the *Pa*DHDPR NLL region is stabilized by internal interactions. The NZ of Lys36 interacts with the Asp40 side chain and the main-chain carboxyl oxygen atom of Ser45. Asp37 interacts with the main-chain nitrogen atoms of Gly39 and Asp40. The residues involved in internal interactions of the NLL are depicted as slate red sticks.

References

1. Shimizu, H. & Shikanai, T. Dihydrodipicolinate reductase-like protein, CRR1, is essential for chloroplast NAD (P) H dehydrogenase in *Arabidopsis*. *The Plant Journal* **52**, 539-547 (2007).

2. Tyagi, V. V., Henke, R. R. & Farkas, W. R. Partial purification and characterization of dihydrodipicolinic Acid reductase from maize. *Plant physiology* **73**, 687-691 (1983).

3. KIMURA, K. A new flavin enzyme catalyzing the reduction of dihydrodipicolinate in sporulating *Bacillus subtilis*: I. Purification and properties. *The Journal of Biochemistry* **77**, 405-413 (1975).

4. Cirilli, M., Zheng, R., Scapin, G. & Blanchard, J. S. The three-dimensional structures of the *Mycobacterium tuberculosis* dihydrodipicolinate reductase− NADH− 2, 6-PDC and− NADPH− 2, 6-PDC complexes. Structural and mutagenic analysis of relaxed nucleotide specificity. *Biochemistry* **42**, 10644-10650 (2003).

5. Dommaraju, S. *et al*. Cloning, expression and crystallization of dihydrodipicolinate reductase from methicillin-resistant *Staphylococcus aureus*. *Acta Crystallographica Section F: Structural Biology and Crystallization Communications* **66**, 57-60 (2010).

6. Pearce, F. G., Sprissler, C. & Gerrard, J. A. Characterization of dihydrodipicolinate reductase from *Thermotoga maritima* reveals evolution of substrate binding kinetics. *Journal of biochemistry* **143**, 617-623 (2008).

7. Ge, X., Olson, A., Cai, S. & Sem, D. S. Binding synergy and cooperativity in dihydrodipicolinate reductase: implications for mechanism and the design of biligand inhibitors. *Biochemistry* **47**, 9966-9980 (2008).

8. Anand, V., Gautam, A., Sareen, D., Singh, T. P. & Tewari, R. Molecular cloning, biochemical and biophysical studies of dihydrodipicolinate reductase of *Pseudomonas aeruginosa* PAO1. *International Journal of Integrative Biology* **11** 145-152 (2011).

9. Schuck, P. Size-distribution analysis of macromolecules by sedimentation velocity ultracentrifugation and lamm equation modeling. *Biophys J*. 78, 1606-1619 (2000).

10. Schuck, P. & Rossmanith, P. Determination of the sedimentation coefficient distribution by least-squares boundary modeling. *Biopolymers*. 54, 328-341 (2000).
